# Persistent One-Year Mortality Gradient After STEMI and COVID-19: Long-Term Outcomes From the NACMI Registry

**DOI:** 10.1016/j.jscai.2026.105395

**Published:** 2026-04-24

**Authors:** Payam Dehghani, Chase J. Ellingson, Jyotpal Singh, Ellen Cravero, G.B. John Mancini, Larissa Stanberry, Mina Madan, Catherine P. Benziger, Nima Ghasemzadeh, Anna E. Bortnick, Rohan Kankaria, Cindy L. Grines, Keshav Nayak, Ehtisham Mahmud, Kevin R. Bainey, M. Chadi Alraies, Akshay Bagai, Rajan A.G. Patel, Shy Amlani, Brian C. Case, Ron Waksman, Jay S. Shavadia, Jay H. Stone, Deepak Acharya, Nosheen Javed, Rodrigo Bagur, Ross Garberich, Santiago Garcia, Timothy D. Henry

**Affiliations:** a Prairie Vascular Research Inc, Regina, Saskatchewan, Canada; b University of Saskatchewan College of Medicine, Regina, Saskatchewan, Canada; c Minneapolis Heart Institute Foundation, Minneapolis, Minnesota; d University of British Columbia, Vancouver, British Columbia, Canada; e Schulich Heart Program, Sunnybrook Health Sciences Centre, University of Toronto, Toronto, Ontario, Canada; f Essentia Health, Duluth, Minnesota; g Georgia Heart Institute, Gainesville, Georgia; h Montefiore Medical Center and Albert Einstein College of Medicine, Bronx, New York; i Johns Hopkins University School of Medicine, Department of Internal Medicine, Baltimore, Maryland; j Northside Hospital Cardiovascular Institute, Atlanta, Georgia; k Scripps Mercy Hospital, San Diego, California; l University of California San Diego, La Jolla, California; m Canadian VIGOUR Centre, University of Alberta, Edmonton, Alberta, Canada; n Detroit Medical Center, Harper University Hospital, Detroit, Michigan; o Terrence Donnelly Heart Center, St Michael’s Hospital, Unity Health Network, University of Toronto, Toronto, Ontario, Canada; p Ochsner Medical Center, New Orleans, Louisiana; University of Virginia, Charlottesville, Virginia; q William Osler Health System, Ontario, Canada; r MedStar Heart & Vascular Institute, Washington, District of Columbia; s Royal University Hospital, Saskatchewan, Canada; t RWJBarnabas Health, West Orange, New Jersey; u University of Arizona, Tucson, Arizona; v Charlton Memorial Hospital, Southcoast Health, Fall River, Massachusetts; w Western University, Ontario, Canada; x The Christ Hospital, Cincinnati, Ohio

**Keywords:** coronavirus, COVID-19, myocardial infarction, STEMI

## Abstract

**Background:**

Patients with COVID-19 and ST-elevation myocardial infarction (STEMI) from the North American COVID-19 Myocardial Infarction (NACMI) registry had elevated in-hospital mortality compared with COVID-19-negative patients and historical controls. We examined 1-year mortality outcomes from the NACMI registry.

**Methods:**

This was a substudy of NACMI centers that participated in long-term follow-up. Patients in the NACMI registry were stratified into COVID-19-positive and COVID-19-negative groups. A historical 2018-2019 control group was derived from the Midwest STEMI Consortium registry. The primary outcome was 1-year mortality.

**Results:**

A total of 2358 STEMI patients (30% female) were included in this study, divided into 3 subgroups: COVID-19-positive (n = 623), COVID-19-negative (n = 694), and historical controls (n = 1041). One-year mortality in COVID-19-positive patients was 45% (HR, 4.88; 95% CI, 3.73-6.39; *P* < .001), compared with 27% (HR, 3.93; 95% CI, 2.92-5.29; *P* < .001) in COVID-19-negative patients and 11% in matched controls (*P* < .001). Most deaths (86%) occurred during the index hospitalization, with a median time to death of 27 days (IQR 6, 343) in the COVID-19-positive group. Among survivors of index hospitalization, 1-year mortality was 12% (COVID-19-positive; HR, 2.20; 95% CI, 1.26-3.85; *P* = .006), 9.6% (COVID-19-negative; HR, 2.31; 95% CI, 1.26-4.21; *P* = .007), and 5.3% (controls) (*P* < .001).

**Conclusions:**

This study describes long-term outcomes in patients with STEMI and COVID-19. We demonstrate that the excess mortality risk associated with COVID-19 STEMI extends beyond the index hospitalization and exhibits a clear risk gradient. The cause is likely multifactorial, including pandemic-era disruptions in care, longer time-to-treatment, and the unique pathophysiology of COVID-19 STEMI.

## Introduction

The North American COVID-19 Myocardial Infarction (NACMI) registry is a prospective, multicenter registry created to characterize the demographics, clinical presentation, management, and outcomes of patients with confirmed or suspected COVID-19 presenting with ST-segment-elevation myocardial infarction (STEMI).^1^ Early NACMI results showed in-hospital mortality rates of 33% among patients with STEMI and COVID-19, compared with 4% among STEMI patients without COVID-19 in the Midwest STEMI Consortium, which served as registry controls.^2^ Elevated in-hospital mortality in STEMI patients with concurrent COVID-19 has also been demonstrated in other cohorts, strengthening the consistency of this observation.^3^^,^^4^

One-year later, NACMI analyses demonstrated temporal changes in clinical characteristics and outcomes among COVID-19 STEMI patients, including a reduction in in-hospital mortality to 23%.^5^ Core laboratory-adjudicated angiographic analysis of STEMI patients with COVID-19 found high-thrombus burden, poor thrombolysis in myocardial infarction (TIMI) flow, frequent stent thrombosis, higher rates of unsuccessful percutaneous coronary intervention (PCI), and multiple culprit arteries in male patients, whereas female patients were more likely to have no clear culprit.^6^ Further, a recent NACMI publication established the electrocardiogram (ECG) as a tool for risk-stratifying patients, demonstrating better prediction of mortality than angiographic analysis.^7^

There has been limited long-term follow-up of COVID-19 STEMI patients; therefore, we present 1-year follow-up all-cause mortality data from the NACMI registry. We hypothesize that COVID-19-positive patients will experience higher mortality rates compared with COVID-19-negative and historical control patients.

## Methods

This substudy involved NACMI sites that collected long-term follow-up data. Patients were eligible for enrollment in the NACMI registry if they were ≥18 years of age, tested positive for COVID-19 or were suspected to have COVID-19, and presented with STEMI or new left-bundle branch block on 12-lead ECG and had clinical suspicion of myocardial ischemia. Further details pertaining to the inclusion criteria can be found in prior NACMI registry work.^1^^,^^2^

Patients were stratified into COVID-19-positive and COVID-19-negative groups. The COVID-19-negative group consisted of patients initially suspected of having COVID-19 (based on symptoms, known COVID-19 exposure, and/or imaging findings) who subsequently tested negative for COVID-19. As in previous reports, a historical control group was included from the Midwest STEMI Consortium,^2^ consisting of patients with follow-up data available from an index hospitalization in 2018 or 2019. In contrast, the COVID-19 groups included patients from 2020 to 2022. Research was carried out in accordance with the applicable ethical guidelines. The requirement for participant consent was waived. Registry design and ethical approval processes have been described previously.^1^

The primary outcome was all-cause mortality at 1 year. Patient characteristics were summarized using counts (%) for categorical and medians (IQR) for continuous variables. The characteristics were compared between groups with Pearson’s χ^2^, Kruskal–Wallis, and Fisher exact tests. The survival functions were estimated using the Kaplan–Meier method, with hospital admission as time zero. Participants who did not experience death were censored at the date of last follow-up or at 1 year (end of study follow up), whichever occurred first. For postdischarge survival, a separate analysis excluded patients who died during the index hospitalization. The proportional Cox regression models were used to estimate the association between the COVID-19 status and the risk of mortality. The models were adjusted for sex, age, race, diabetes mellitus, reperfusion strategy (PCI), length of hospitalization, and stratified by cardiogenic shock pre-PCI. The proportional hazards assumption was verified on Schoenfeld residual analysis in both models. The model estimates are reported with their 95% CI and *P* values. The analysis was performed in R v 4.5 (R Core Team) within RStudio (v2026.01, Posit Software, PBC) environment.

## Results

A total of 2358 patients (30% female patients) were included in this study (Table 1); participants were stratified into the following groups: COVID-19-positive (n = 623), COVID-19-negative (n = 694), and historical controls (n = 1041). Across all participants, age distribution (*P* = .3) was as follows: 18 to 55 (25%), 56 to 65 (28%), 66 to 75 (25%), 76 to 85 (16%), and >85 years (5.4%). Compared with controls, COVID-19-positive patients had a longer median length of stay (6 vs 2 days; *P* < .001) and were less often white (55% vs 91%; *P* < .001). They had a lower prevalence of prior myocardial infarction (15% vs 22%; *P* < .001), dyslipidemia (46% vs 56%; *P* < .001), and current smoking (20% vs 28%; *P* < .001). In contrast, hypertension (69% vs 64%; *P* < .001) and diabetes mellitus (43% vs 26%; *P* < .001) were more prevalent among COVID-19-positive patients. Additional details on participant demographics are provided in Table 1.

**Table 1:** Participant characteristics

Characteristic	Overall (N = 2358)	Control (n = 1041)	COVID-19-negative (n = 694)	COVID-19-positive (n = 623)	*P* value
Biological sex					.40
Female	710 (30)	328 (32)	200 (29)	182 (29)	
Male	1648 (70)	713 (68)	494 (71)	441 (71)	
Age, y					.30
18-55	585 (25)	260 (25)	179 (26)	146 (23)	
56-65	659 (28)	270 (26)	195 (28)	194 (31)	
66-75	599 (25)	267 (26)	180 (26)	152 (24)	
76-85	380 (16)	180 (17)	97 (14)	103 (17)	
>85	127 (5.4)	58 (5.6)	42 (6.1)	27 (4.3)	
Race/ethnicity					<.001
Black	177 (8.7)	73 (7.1)	38 (8.8)	66 (12)	
Asian	57 (2.8)	6 (0.6)	15 (3.5)	36 (6.3)	
White	1597 (79)	940 (91)	342 (79)	315 (55)	
Hispanic	120 (5.9)	2 (0.2)	19 (4.4)	99 (17)	
Indigenous	23 (1.1)	0 (0)	9 (2.1)	14 (2.5)	
Other	58 (2.9)	10 (1.0)	8 (1.9)	40 (7.0)	
BMI, kg/m^2^	29 (25, 33)	29 (26, 33)	29 (25, 33)	28 (25, 33)	.10
History of CAD	661 (29)	337 (32)	171 (26)	153 (26)	.005
Previous PCI	486 (21)	276 (27)	120 (18)	90 (16)	<.001
Previous MI	432 (19)	232 (22)	117 (18)	83 (15)	<.001
Hypertension	1564 (68)	662 (64)	483 (73)	419 (69)	<.001
Dyslipidemia	1230 (54)	582 (56)	376 (59)	272 (46)	<.001
DM	765 (33)	269 (26)	239 (36)	257 (43)	<.001
Previous stroke/TIA	199 (8.8)	77 (7.4)	65 (9.9)	57 (9.9)	.12
Smoker status					<.001
Current	652 (29)	290 (28)	244 (39)	118 (20)	
Former	665 (30)	348 (33)	156 (25)	161 (28)	
Never	935 (42)	402 (39)	232 (37)	301 (52)	
Cardiac arrest					<.001
Pre-PCI	286 (13)	121 (12)	105 (16)	60 (11)	
In cath	41 (1.8)	21 (2.0)	8 (1.2)	12 (2.1)	
Post-PCI	76 (3.3)	17 (1.6)	29 (4.3)	30 (5.3)	
None	1880 (82)	882 (85)	533 (79)	465 (82)	
Cardiogenic shock					.003
Pre-PCI	343 (15)	147 (14)	110 (17)	86 (15)	
In cath	46 (2.0)	10 (1.0)	22 (3.3)	14 (2.5)	
Post-PCI	115 (5.1)	64 (6.1)	23 (3.5)	28 (5.0)	
None	1763 (78)	820 (79)	511 (77)	432 (77)	
D2B, min	56 (31, 97)	40 (25, 71)	79 (53, 130)	73 (45, 124)	<.001
Ejection fraction, %	49 (37, 58)	52 (40, 58)	45 (35, 55)	45 (34, 55)	<.001
Reperfusion strategy					<.001
Primary PCI	1630 (72)	749 (77)	533 (77)	348 (59)	
Medical therapy	158 (7.0)	—	63 (9.1)	95 (16)	
CABG	36 (1.6)	—	29 (4.2)	7 (1.2)	
Facilitated/rescue PCI	24 (1.1)	—	11 (1.6)	13 (2.2)	
Thrombolytics	73 (3.2)	—	38 (5.5)	35 (5.9)	
No angiogram performed	110 (4.9)	—	15 (2.2)	95 (16)	
Other	225 (10.0)	225 (23)	0 (0)	0 (0)	
Year of presentation					
2018	476 (33)	476 (46)	0 (0)	0 (0)	
2019	565 (39)	565 (54)	0 (0)	0 (0)	
2020	171 (12)	0 (0)	12 (19)	159 (46)	
2021	207 (14)	0 (0)	52 (81)	155 (45)	
2022	29 (2.0)	0 (0)	0 (0)	29 (8.5)	
Length of stay, d	3 (2, 7)	2 (2, 5)	3 (2, 6)	6 (2, 12)	<.001

Values are presented as n (%) or median (Q1, Q3). Pearson’s χ^2^ test, Kruskal–Wallis rank sum test, and Fisher exact test were used.

BMI, body mass index; CABG, coronary artery bypass grafting; CAD, coronary artery disease; DM, diabetes mellitus; D2B, door-to-balloon; MI, myocardial infarction; PCI, percutaneous coronary intervention; TIA, transient ischemic attack.

Door-to-balloon times were longer (*P* < .001) for both COVID-19-positive (73 [45, 124] minutes) and negative (79 [53, 130] minutes) patients than for controls (40 [25, 71] minutes). Cardiogenic shock occurred in 23% of COVID-19-positive and negative participants, compared with 21% in controls. Reperfusion strategies also differed, with only 59% of COVID-19-positive patients undergoing primary PCI, compared with 77% in both the COVID-19-negative and control groups (*P* < .001). Furthermore, 16% of the COVID-19-positive group received medical management alone, compared with only 9.1% in the COVID-19-negative group.

All-cause mortality was significantly higher in the COVID-19-positive (HR, 4.88; 95% CI, 3.73-6.39; *P* < .001) and COVID-19-negative (HR, 3.93; 95% CI, 2.92-5.29; *P* < .001) groups compared with controls (Central Illustration, Table 2). One-year mortality rates were 45% (COVID-19-positive), 27% (COVID-19-negative), and 11% (controls) (*P* < .001) (Table 3). The median time to death was shortest in the COVID-19-positive group (27 [6, 343] days) compared with the COVID-19-negative group (75 [6, 336] days) (*P* < .001). Most deaths in COVID-19-positive (84%) and COVID-19-negative (71%) patients occurred during the index hospitalization, compared with controls (44%).

**Central Illustration:**
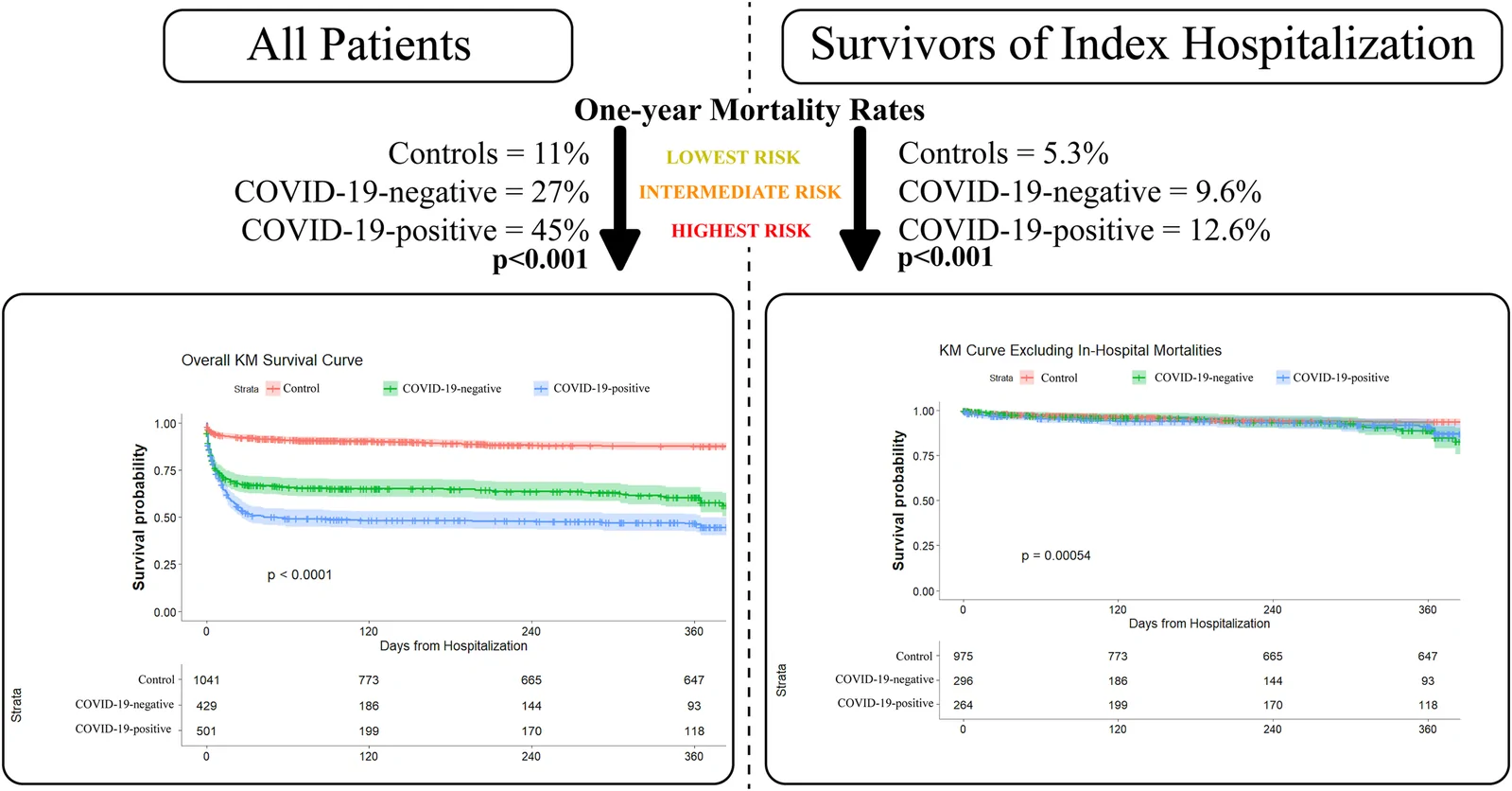
**One-yearall-causemortality rates and Kaplan–Meier survival curves.** The left side represents the entire cohort, whereas the right side excludes those who died during index hospitalization. HR, hazard ratio; KM, Kaplan–Meier.

**Table 2:** Cox regression modeling for all-cause mortality

	Hazard ratio	95% CI	*P* value
All patients in Cox regression modeling (n = 1592)			
Group			
Control	—	—	
COVID-19 negative	3.93	2.92-5.29	<.001
COVID-19 positive	4.88	3.73-6.39	<.001
Male sex	0.88	0.71-1.09	.20
Age >65 y	1.52	1.22-1.88	<.001
Race/ethnicity: White	0.78	0.62-0.99	.043
Diabetes mellitus	1.40	1.13-1.73	.002
Reperfusion strategy - PCI	0.49	0.40-0.61	<.001
Survivors of index hospitalization (n = 1299)			
Group			
Control	—	—	
COVID-19 negative	2.31	1.26-4.21	.007
COVID-19 positive	2.20	1.26-3.85	.006
Male sex	0.61	0.39-0.96	.034
Age >65 y	2.03	1.27-3.27	.003
Race/ethnicity: White	1.02	0.55-1.90	>.99
Diabetes mellitus	1.25	0.78-2.00	.40
Reperfusion strategy - PCI	0.63	0.39-1.02	.062
Length of hospitalization, d	1.02	1.01-1.03	.002

PCI, percutaneous coronary intervention.

**Table 3:** Assessment of all-cause mortality

	Total	Control	COVID-19-negative	COVID-19-positive	*P* value
All patients	N = 2358	n = 1041	n = 694	n = 623	
One-year mortality	588 (25%)	118 (11%)	187 (27%)	283 (45%)	<.001
In-hospital mortality	436 (18%)	66 (6.3%)	133 (19%)	237 (38%)	<.001
Time to death, d	232 (22, 365)	365 (114, 365)	75 (6, 336)	27 (6, 343)	<.001
Missing	387	0	265	122	
Survivors of index hospitalization	N = 1922	n = 975	n = 561	n = 386	
One-year mortality	152 (7.9%)	52 (5.3%)	54 (9.6%)	46 (12%)	<.001
Time to death, d	365 (123, 365)	365 (162, 365)	218 (61, 365)	336 (121, 365)	<.001
Missing	387	0	265	122	
Length of hospitalization, d	3 (2, 6)	2 (2, 5)	3 (2, 6)	5 (2, 10)	<.001
Missing	18	1	7	10	

Values are presented as n (%) or median (Q1, Q3). Pearson’s χ^2^ test; Kruskal–Wallis rank sum test.

Among patients who survived index hospitalization, the risk of mortality remained elevated in COVID-19-positive (HR, 2.20; 95% CI, 1.26-3.85; *P* = .006) and negative (HR, 2.31; 95% CI, 1.26-4.21; *P* = .007) patients (Central Illustration, Table 2). One-year mortality rates in these surviving patients were 12% (COVID-19-positive), 9.6% (COVID-19 negative), and 5.3% (controls) (*P* < .001) (Table 3).

The missing data in this cohort are highlighted in Supplemental Table S1. Given the limited follow-up outcome data, we also summarized the data by those with no missing outcome data versus those with missing outcome data (Supplementary Table S2). From the covariates in the models, race/ethnicity had the largest degree of missingness; accordingly, we conducted a sensitivity analysis and estimated the risk of mortality excluding race/ethnicity (Supplementary Table S3). Importantly, this did not impact the model estimates.

## Discussion

This analysis fills a key evidence gap by reporting 1-year outcomes in STEMI patients with COVID-19. Consistent with previous reports,^2^^,^^6^ we found that in-hospital mortality is markedly higher in COVID-19 STEMI patients. The key new finding is that excess risk persists beyond discharge. After excluding in-hospital deaths, 1-year all-cause mortality remained significantly elevated, with a clear gradient across groups: highest in COVID-19-positive patients, intermediate in COVID-19-negative patients, and lowest in historical controls.

Several factors may explain this persistent mortality gradient. Both COVID-19-positive and COVID-19-negative patients experienced substantially longer door-to-balloon times than historical controls, reflecting pandemic-era delays in presentation and care delivery. Prolonged ischemic time is strongly associated with larger infarct size and worse long-term outcomes.^2^^,^^8^ During the COVID-19 pandemic, STEMI patients often presented later to hospital,^2^^,^^3^^,^^9^ leading to longer ischemic times, larger infarcts, and worse outcomes.^10^ Notably, one study demonstrated significant increases in symptom-to-admission times for COVID-19-positive STEMI patients (339 minutes) compared with pre-COVID-19 reference data (173 minutes) (*P* < .001).^3^ The intermediate outcomes observed in COVID-19-negative patients suggest that system-level disruptions during the pandemic, beyond confirmed infection alone, adversely affected STEMI prognosis.

In addition, prior NACMI core laboratory analyses demonstrated a prothrombotic and high-thrombus burden angiographic phenotype in COVID-19-positive STEMI, including lower TIMI flow, more frequent unsuccessful PCI, and more thrombotic lesions,^6^ which may contribute to greater residual myocardial injury, microvascular dysfunction, and downstream risk. However, as demonstrated in previous publications, fewer culprit arteries are identified in COVID-19 STEMI patients, and there is a higher rate of microvascular dysfunction,^2^^,^^10^ which may partly explain the suboptimal PCI outcomes.^6^ In short, pandemic-era disruptions in care and longer ischemic times likely increase mortality risk in both COVID-19-era groups; however, we propose that the additional risk in the COVID-19-positive group is due to the unique angiographic phenotype of COVID-19 STEMI.

Prior analyses suggest that lower rates of PCI in patients with STEMI and COVID-19 reflect a distinct disease phenotype rather^10^ than differences in treatment approach. This phenotype is characterized by a lower frequency of culprit lesions on angiography, higher thrombus burden, and higher incidence of microvascular obstruction^11^ and myocardial infarction with nonobstructive coronary arteries.^12^ Consistent with this pathophysiology, NACMI angiographic analyses demonstrate lower procedural success, with reduced rates of post-PCI TIMI 3 flow and a higher incidence of unsuccessful PCI driven by persistent thrombus burden,^6^ reinforcing the concept of a uniquely thrombotic and microvascular-predominant STEMI phenotype in COVID-19.^10^ In addition, a subset of patients, particularly early in the pandemic, presented with severe respiratory compromise requiring intensive care and mechanical ventilation,^2^^,^^13^ further reducing the feasibility of invasive management.

The interpretation of these findings warrants several considerations. First, the use of all-cause mortality precludes attribution of deaths specifically to the index STEMI event, COVID-19-related causes, or other causes, thereby limiting mechanistic inference. Second, the excess mortality observed in COVID-19-positive patients is unlikely to be explained by differences in revascularization, as prior research suggests that such differences are due to the lack of an identifiable culprit artery.^2^ Rather, the excess mortality likely reflects a distinct pathophysiologic phenotype of STEMI in the setting of COVID-19, characterized by diffuse thrombotic burden and microvascular involvement, in which epicardial culprit lesions may be less clearly identifiable and less amenable to conventional revascularization strategies. Importantly, COVID-19 itself confers an excess mortality risk beyond the acute presentation; in a large, risk-matched cohort, overall 2-year mortality remained higher among individuals with prior COVID-19 infection than among uninfected controls, even outside the context of STEMI.^14^ Taken together, these findings suggest that the higher mortality observed in COVID-19-positive STEMI patients likely reflects both the system-level impact of the COVID-19 pandemic and a fundamentally different thrombo-inflammatory disease process.

These findings have practical implications. STEMI patients presenting during COVID-era care conditions remain at increased risk even after surviving hospitalization, particularly those with confirmed infection. Postdischarge risk does not return to that of historical STEMI cohorts, supporting the need for closer follow-up and intensified secondary prevention in these higher-risk groups.

Several limitations should be acknowledged. With the use of all-cause mortality, it is possible that some of the excess deaths in the COVID-19-positive group are a result of their infection, independent of the STEMI; nonetheless, it still remains true that COVID-19 STEMI is a higher-risk cohort, requiring close monitoring and follow-up. The lack of cause-of-death adjudication is another limitation in the interpretation of our results. Data were missing for certain demographic and outcome variables (Supplementary Table S1). Only a subset of NACMI sites agreed to collect data on long-term outcomes; therefore, 1-year outcome data were missing for 387 patients. Still, the magnitude and consistency of mortality across analyses support the association between COVID-19 status and mortality. It is also important to note that the historical control group was from the 2 years preceding the pandemic, and there may have been other system-related differences aside from the pandemic that could confound comparisons with this group. One must also consider that the COVID-19-negative group was suspected of having COVID-19 initially, but subsequently tested negative. These patients had longer total ischemic times and were more complex than a contemporary STEMI-only cohort. For time-to-event outcomes, the admission date was approximated as the first day of the respective quarter, which may bias analyses toward overestimating time to subsequent events and thereby affect Kaplan–Meier estimates.

## Conclusion

This NACMI analysis demonstrates that excess mortality in the COVID-19-era STEMI extends beyond the index hospitalization. One-year mortality shows a clear gradient, highest in COVID-19-positive patients, intermediate in COVID-19-negative patients, and lowest in historical controls. Although early in-hospital deaths account for much of the risk, mortality remains elevated among hospital survivors. These findings extend prior NACMI reports and identify persistent long-term risk in COVID-19-associated STEMI.

## CRediT authorship contribution statement

**Payam Dehghani:** Conceptualization, Funding acquisition, Investigation, Methodology, Writing – original draft. **Chase J. Ellingson:** Visualization, Writing – original draft. **Jyotpal Singh:** Writing – review & editing. **Ellen Cravero:** Formal analysis, Writing – review & editing. **G.B. John Mancini:** Investigation, Writing – review & editing. **Larissa Stanberry:** Formal analysis, Writing – review & editing. **Mina Madan:** Investigation, Writing – review & editing. **Catherine P. Benziger:** Investigation, Writing – review & editing. **Nima Ghasemzadeh:** Investigation, Writing – review & editing. **Anna E. Bortnick:** Investigation, Writing – review & editing. **Rohan Kankaria:** Investigation, Writing – review & editing. **Cindy L. Grines:** Investigation, Writing – review & editing. **Keshav Nayak:** Investigation, Writing – review & editing. **Ehtisham Mahmud:** Investigation, Writing – review & editing. **Kevin R. Bainey:** Investigation, Writing – review & editing. **M. Chadi Alraies:** Investigation, Writing – review & editing. **Akshay Bagai:** Investigation, Writing – review & editing. **Rajan A.G. Patel:** Investigation, Writing – review & editing. **Shy Amlani:** Investigation, Writing – review & editing. **Brian C. Case:** Investigation, Writing – review & editing. **Ron Waksman:** Investigation, Writing – review & editing. **Jay S. Shavadia:** Investigation, Writing – review & editing. **Jay H. Stone:** Investigation, Writing – review & editing. **Deepak Acharya:** Investigation, Writing – review & editing. **Nosheen Javed:** Investigation, Writing – review & editing. **Rodrigo Bagur:** Investigation, Writing – review & editing. **Ross Garberich:** Investigation, Writing – review & editing. **Santiago Garcia:** Investigation, Writing – review & editing. **Timothy D. Henry:** Conceptualization, Funding acquisition, Investigation, Methodology, Project administration, Resources, Writing – review & editing.
