# Supplementary material for: Persistent One-Year Mortality Gradient After STEMI and COVID-19: Long-Term Outcomes From the NACMI Registry
Source: J Soc Cardiovasc Angiogr Interv. 2026 Apr 24;5(8):105395. doi: 10.1016/j.jscai.2026.105395 (PMC13491345; doi:10.1016/j.jscai.2026.105395)
Supplement: Supplementary file 1 [file mmc1.docx]

**Supplementary Table S1:** Participant characteristics (including degree of missing data)

| Characteristic | Overall  (n=2358) | Control (n=1041) | COVID-19-negative  (n= 694) | COVID-19-positive  (n=623) | p-value |
| --- | --- | --- | --- | --- | --- |
| Biological Sex |  |  |  |  | 0.4 |
| Female | 710 (30%) | 328 (32%) | 200 (29%) | 182 (29%) |  |
| Male | 1648 (70%) | 713 (68%) | 494 (71%) | 441 (71%) |  |
| Age |  |  |  |  | 0.3 |
| 18-55 | 585 (25%) | 260 (25%) | 179 (26%) | 146 (23%) |  |
| 56-65 | 659 (28%) | 270 (26%) | 195 (28%) | 194 (31%) |  |
| 66-75 | 599 (25%) | 267 (26%) | 180 (26%) | 152 (24%) |  |
| 76-85 | 380 (16%) | 180 (17%) | 97 (14%) | 103 (17%) |  |
| >85 | 127 (5.4%) | 58 (5.6%) | 42 (6.1%) | 27 (4.3%) |  |
| Missing | 8 | 6 | 1 | 1 |  |
| Race/Ethnicity |  |  |  |  |  |
| Black | 177 (8.7%) | 73 (7.1%) | 38 (8.8%) | 66 (12%) |  |
| Asian | 57 (2.8%) | 6 (0.6%) | 15 (3.5%) | 36 (6.3%) |  |
| White | 1597 (79%) | 940 (91%) | 342 (79%) | 315 (55%) |  |
| Hispanic | 120 (5.9%) | 2 (0.2%) | 19 (4.4%) | 99 (17%) |  |
| Indigenous | 23 (1.1%) | 0 (0%) | 9 (2.1%) | 14 (2.5%) |  |
| Other | 58 (2.9%) | 10 (1.0%) | 8 (1.9%) | 40 (7.0%) |  |
| Missing | 326 | 10 | 263 | 53 |  |
| White | 1597 (79%) | 940 (91%) | 342 (79%) | 315 (55%) | <0.001 |
| Missing | 326 | 10 | 263 | 53 |  |
| BMI | 29 (25, 33) | 29 (26, 33) | 29 (25, 33) | 28 (25, 33) | 0.10 |
| Missing | 134 | 21 | 68 | 45 |  |
| History of CAD | 661 (29%) | 337 (32%) | 171 (26%) | 153 (26%) | 0.005 |
| Missing | 79 | 0 | 35 | 44 |  |
| Previous PCI | 486 (21%) | 276 (27%) | 120 (18%) | 90 (16%) | <0.001 |
| Missing | 80 | 0 | 28 | 52 |  |
| Previous MI | 432 (19%) | 232 (22%) | 117 (18%) | 83 (15%) | <0.001 |
| Missing | 99 | 5 | 38 | 56 |  |
| HTN | 1564 (68%) | 662 (64%) | 483 (73%) | 419 (69%) | <0.001 |
| Missing | 51 | 0 | 35 | 16 |  |
| DLD | 1230 (54%) | 582 (56%) | 376 (59%) | 272 (46%) | <0.001 |
| Missing | 97 | 3 | 57 | 37 |  |
| DM | 765 (33%) | 269 (26%) | 239 (36%) | 257 (43%) | <0.001 |
| Missing | 59 | 3 | 26 | 30 |  |
| Previous Stroke/TIA | 199 (8.8%) | 77 (7.4%) | 65 (9.9%) | 57 (9.9%) | 0.12 |
| Missing | 84 | 2 | 36 | 46 |  |
| Smoker Status |  |  |  |  | <0.001 |
| Current | 652 (29%) | 290 (28%) | 244 (39%) | 118 (20%) |  |
| Former | 665 (30%) | 348 (33%) | 156 (25%) | 161 (28%) |  |
| Never | 935 (42%) | 402 (39%) | 232 (37%) | 301 (52%) |  |
| Missing | 106 | 1 | 62 | 43 |  |
| Cardiac Arrest |  |  |  |  | <0.001 |
| Pre-PCI | 286 (13%) | 121 (12%) | 105 (16%) | 60 (11%) |  |
| In Cath | 41 (1.8%) | 21 (2.0%) | 8 (1.2%) | 12 (2.1%) |  |
| Post-PCI | 76 (3.3%) | 17 (1.6%) | 29 (4.3%) | 30 (5.3%) |  |
| None | 1880 (82%) | 882 (85%) | 533 (79%) | 465 (82%) |  |
| Missing | 75 | 0 | 19 | 56 |  |
| Cardiogenic Shock |  |  |  |  | 0.003 |
| Pre-PCI | 343 (15%) | 147 (14%) | 110 (17%) | 86 (15%) |  |
| In Cath | 46 (2.0%) | 10 (1.0%) | 22 (3.3%) | 14 (2.5%) |  |
| Post-PCI | 115 (5.1%) | 64 (6.1%) | 23 (3.5%) | 28 (5.0%) |  |
| None | 1763 (78%) | 820 (79%) | 511 (77%) | 432 (77%) |  |
| Missing | 91 | 0 | 28 | 63 |  |
| D2B (minutes) | 56 (31, 97) | 40 (25, 71) | 79 (53, 130) | 73 (45, 124) | <0.001 |
| Missing | 729 | 131 | 248 | 350 |  |
| Ejection Fraction % | 49 (37, 58) | 52 (40, 58) | 45 (35, 55) | 45 (34, 55) | <0.001 |
| Missing | 517 | 64 | 203 | 250 |  |
| Reperfusion strategy |  |  |  |  | <0.001 |
| Primary PCI | 1630 (72%) | 749 (77%) | 533 (77%) | 348 (59%) |  |
| Medical therapy | 158 (7.0%) | - | 63 (9.1%) | 95 (16%) |  |
| CABG | 36 (1.6%) | - | 29 (4.2%) | 7 (1.2%) |  |
| Facilitated/Rescue PCI | 24 (1.1%) | - | 11 (1.6%) | 13 (2.2%) |  |
| Thrombolytics | 73 (3.2%) | - | 38 (5.5%) | 35 (5.9%) |  |
| No angiogram performed | 110 (4.9%) | - | 15 (2.2%) | 95 (16%) |  |
| Other | 225 (10.0%) | 225 (23%) | 0 (0%) | 0 (0%) |  |
| Missing | 102 | 67 | 5 | 30 |  |
| Year of Presentation |  |  |  |  |  |
| 2018 | 476 (33%) | 476 (46%) | 0 (0%) | 0 (0%) |  |
| 2019 | 565 (39%) | 565 (54%) | 0 (0%) | 0 (0%) |  |
| 2020 | 171 (12%) | 0 (0%) | 12 (19%) | 159 (46%) |  |
| 2021 | 207 (14%) | 0 (0%) | 52 (81%) | 155 (45%) |  |
| 2022 | 29 (2.0%) | 0 (0%) | 0 (0%) | 29 (8.5%) |  |
| Missing | 910 | 0 | 630 | 280 |  |
| Length of Stay (days) | 3 (2, 7) | 2 (2, 5) | 3 (2, 6) | 6 (2, 12) | <0.001 |
| Missing | 30 | 1 | 11 | 18 |  |
| Note: Data is presented as n (%); Median (Q1, Q3). Pearson's Chi-squared test, Kruskal-Wallis rank sum test, and Fisher's exact test were used. Abbreviations: BMI = body mass index, CABG = coronary artery bypass grafting, CAD = coronary artery disease, DLD = dyslipidemia, DM = diabetes mellitus, D2B = door-to-balloon, EF = ejection fraction, HTN = hypertension, Hx = history, MI = myocardial infarction, PCI = percutaneous coronary intervention, TIA = transient ischemic attack. **Supplementary Table S2:** Participant characteristics stratified by missing outcome data (NACMI participants only) Characteristic \| Overall (n=1317) \| No missing outcome data (n=930) \| Missing outcome data (n=387) \| p-value; Biological Sex \| \| \| \| 0.057; Female \| 382 (29%) \| 284 (31%) \| 98 (25%) \|; Male \| 935 (71%) \| 646 (69%) \| 289 (75%) \|; Age \| \| \| \| 0.036; 18-55 \| 325 (25%) \| 215 (23%) \| 110 (28%) \|; 56-65 \| 389 (30%) \| 266 (29%) \| 123 (32%) \|; 66-75 \| 332 (25%) \| 248 (27%) \| 84 (22%) \|; 76-85 \| 200 (15%) \| 153 (16%) \| 47 (12%) \|; >85 \| 69 (5.2%) \| 47 (5.1%) \| 22 (5.7%) \|; Missing \| 2 \| 1 \| 1 \|; Race/Ethnicity \| \| \| \| 0.001; African American \| 104 (10%) \| 83 (11%) \| 21 (8.4%) \|; Asian \| 51 (5.1%) \| 44 (5.9%) \| 7 (2.8%) \|; Caucasian \| 657 (66%) \| 467 (62%) \| 190 (76%) \|; Hispanic \| 118 (12%) \| 98 (13%) \| 20 (8.0%) \|; Indigenous \| 23 (2.3%) \| 16 (2.1%) \| 7 (2.8%) \|; Other \| 48 (4.8%) \| 43 (5.7%) \| 5 (2.0%) \|; Missing \| 316 \| 179 \| 137 \|; White \| 657 (66%) \| 467 (62%) \| 190 (76%) \| <0.001; Missing \| 316 \| 179 \| 137 \|; BMI \| 29 (25, 33) \| 29 (25, 33) \| 29 (25, 33) \| 0.6; Missing \| 113 \| 79 \| 34 \|; History of CAD \| 324 (26%) \| 230 (27%) \| 94 (25%) \| 0.5; Missing \| 79 \| 71 \| 8 \|; Previous PCI \| 210 (17%) \| 145 (17%) \| 65 (17%) \| >0.9; Missing \| 80 \| 76 \| 4 \|; Previous MI \| 200 (16%) \| 135 (16%) \| 65 (17%) \| 0.6; Missing \| 94 \| 86 \| 8 \|; Hypertension \| 902 (71%) \| 645 (72%) \| 257 (68%) \| 0.14; Missing \| 51 \| 40 \| 11 \|; Dyslipidemia \| 648 (53%) \| 444 (52%) \| 204 (55%) \| 0.3; Missing \| 94 \| 77 \| 17 \|; Diabetes \| 496 (39%) \| 372 (42%) \| 124 (32%) \| <0.001; Missing \| 56 \| 52 \| 4 \|; Previous Stroke/TIA \| 122 (9.9%) \| 91 (11%) \| 31 (8.2%) \| 0.2; Missing \| 82 \| 74 \| 8 \|; Smoker Status \| \| \| \| <0.001; Current \| 362 (30%) \| 224 (26%) \| 138 (38%) \|; Former \| 317 (26%) \| 226 (27%) \| 91 (25%) \|; Never \| 533 (44%) \| 398 (47%) \| 135 (37%) \|; Missing \| 105 \| 82 \| 23 \|; Cardiac Arrest \| \| \| \| <0.001; In Cath \| 20 (1.6%) \| 17 (2.0%) \| 3 (0.8%) \|; None \| 998 (80%) \| 661 (77%) \| 337 (89%) \|; Post-PCI \| 59 (4.8%) \| 55 (6.4%) \| 4 (1.1%) \|; Pre-PCI \| 165 (13%) \| 129 (15%) \| 36 (9.5%) \|; Missing \| 75 \| 68 \| 7 \|; Cardiogenic Shock \| \| \| \| <0.001; In Cath \| 36 (2.9%) \| 32 (3.8%) \| 4 (1.1%) \|; None \| 943 (77%) \| 609 (72%) \| 334 (88%) \|; Post-PCI \| 51 (4.2%) \| 40 (4.7%) \| 11 (2.9%) \|; Pre-PCI \| 196 (16%) \| 166 (20%) \| 30 (7.9%) \|; Missing \| 91 \| 83 \| 8 \|; D2B (minutes) \| 78 (50, 128) \| 78 (51, 120) \| 79 (50, 143) \| 0.5; Missing \| 598 \| 456 \| 142 \|; Ejection Fraction % \| 45 (35, 55) \| 45 (33, 55) \| 50 (38, 58) \| <0.001; Missing \| 453 \| 339 \| 114 \|; Reperfusion Strategy \| \| \| \| <0.001; CABG \| 36 (2.8%) \| 18 (2.0%) \| 18 (4.8%) \|; Facilitated/Rescue PCI \| 24 (1.9%) \| 18 (2.0%) \| 6 (1.6%) \|; Medical therapy \| 158 (12%) \| 131 (14%) \| 27 (7.2%) \|; No angiogram \| 110 (8.6%) \| 96 (11%) \| 14 (3.7%) \|; Primary PCI \| 881 (69%) \| 605 (67%) \| 276 (73%) \|; Thrombolytics \| 73 (5.7%) \| 37 (4.1%) \| 36 (9.5%) \|; Missing \| 35 \| 25 \| 10 \|; Year of Presentation \| \| \| \| >0.9; 2020 \| 171 (42%) \| 159 (42%) \| 12 (44%) \|; 2021 \| 207 (51%) \| 193 (51%) \| 14 (52%) \|; 2022 \| 29 (7.1%) \| 28 (7.4%) \| 1 (3.7%) \|; Missing \| 910 \| 550 \| 360 \|; Length of Stay (days) \| 4 (2, 9) \| 4 (2, 10) \| 3 (2, 7) \| 0.049; Missing \| 29 \| 23 \| 6 \|; Note: Data is presented as n (%); Median (Q1, Q3). Pearson's Chi-squared test; Wilcoxon rank sum test; Fisher's exact test. Abbreviations: BMI = body mass index, CABG = coronary artery bypass grafting, CAD = coronary artery disease, DLD = dyslipidemia, DM = diabetes mellitus, D2B = door-to-balloon, EF = ejection fraction, HTN = hypertension, Hx = history, MI = myocardial infarction, PCI = percutaneous coronary intervention, TIA = transient ischemic attack. \| \| \| \|; **Supplementary Table S3:** Cox regression modelling for all-cause mortality excluding race/ethnicity (All Patients (n=1758) \| \| \|; \| HR \| 95% CI \| p-value; Group \| \| \|; Control \| -- \| -- \|; COVID-19-negative \| 3.91 \| 3.00, 5.10 \| <0.001; COVID-19-positive \| 5.51 \| 4.30, 7.06 \| <0.001; Male \| 0.83 \| 0.68, 1.02 \| 0.075; Age >65 \| 1.57 \| 1.28, 1.91 \| <0.001; DM \| 1.36 \| 1.11, 1.65 \| 0.002; Reperfusion strategy - PCI \| 0.57 \| 0.46, 0.69 \| <0.001; Survivors of Index Hospitalization (n=1299) \| \| \|; \| HR \| 95% CI \| p-value; Group \| \| \|; Control \| -- \| -- \|; COVID-19-negative \| 2.51 \| 1.49, 4.20 \| <0.001; COVID-19-positive \| 2.11 \| 1.24, 3.57 \| 0.006; Male \| 0.62 \| 0.41, 0.96 \| 0.031; Age >65 \| 2.06 \| 1.33, 3.19 \| 0.001; DM \| 1.27 \| 0.82, 1.96 \| 0.3; Reperfusion strategy - PCI \| 0.61 \| 0.39, 0.95 \| 0.030; Length of Hospitalization (days) \| 1.02 \| 1.01, 1.03 \| 0.001) Abbreviations: CI = confidence interval, DM = diabetes mellitus, HR = hazard ratio, PCI = percutaneous coronary intervention \| \| \| \| | | | | | |
